# Effectiveness of Dry Needling Combined With Exercise Versus Exercise Alone in Various Tendinopathies: A Systematic Review and Meta-Analysis

**DOI:** 10.7759/cureus.92833

**Published:** 2025-09-21

**Authors:** Muhammad Tayyab, Zawar Ahmad, Muhammad Tanveer, Mahmood Ahmad, Rizwan Akbar, Suleman Shah, Rahman Syed, Ameer Afzal Khan, Anfal Khan, Mohsin Ali, Muhammad Shabir

**Affiliations:** 1 Trauma and Orthopaedics, Bradford Teaching Hospitals, Bradford, GBR; 2 Trauma and Orthopaedics, Kettering General Hospital, Kettering, GBR; 3 Trauma and Orthopaedics, University Hospital of North Midlands/Royal Stoke University Hospital, Stoke-on-Trent, GBR; 4 Trauma and Orthopaedics, Milton Keynes University Hospital, Milton Keynes, GBR; 5 Trauma and Orthopaedics, University College London Hospital, London, GBR; 6 Nursing, Fatima College of Health Sciences, Al Ain, ARE; 7 Internal Medicine, Swat Medical College, Swat, PAK; 8 Internal Medicine, Saidu Medical College, Swat, PAK; 9 Orthopaedics, Saidu Group of Teaching Hospitals, Swat, PAK

**Keywords:** dry needling, dry needling plus exercise, exercise-based therapy, systematic review and meta-analysis, tendinopathy

## Abstract

This systematic review and meta-analysis evaluated the effectiveness of dry needling combined with exercise compared with exercise alone for pain and functional outcomes in tendinopathies. A systematic search of PubMed, Cochrane Library, ClinicalTrials.gov, and Google Scholar was conducted from inception to August 13, 2025, for randomized controlled trials involving adults with clinically confirmed tendinopathy. Eligible studies compared dry needling plus exercise with exercise alone and reported pain or functional outcomes. Four trials with 255 patients were included, covering lateral epicondylitis (two trials), patellar tendinopathy (one trial), and rotator cuff tendinopathy (one trial). Pooled analysis showed that dry needling combined with exercise reduced pain more than exercise alone (mean difference (MD) = −2.14; 95% CI, −3.50 to −0.78), although heterogeneity was substantial (I² = 95%). Immediate-term pain reduction was modest (MD = −0.44; 95% CI, −0.87 to 0.00), with larger but variable effects at mid- and long-term follow-ups. Functional outcomes favored the combined intervention, particularly for Patient-Rated Tennis Elbow Evaluation scores at 3-4 weeks (MD = −5.24; 95% CI, −6.89 to −3.60; I² = 21%). Risk of bias was low in one trial and raised some concerns in three, mainly due to blinding and outcome assessment limitations. Overall, dry needling combined with exercise appears to provide greater pain relief and functional improvement than exercise alone, especially in the short to long term. However, substantial heterogeneity and methodological concerns highlight the need for further high-quality randomized controlled trials to confirm these findings and establish optimal treatment protocols.

## Introduction and background

Tendinopathies are among the most prevalent musculoskeletal disorders, accounting for a significant proportion of sports- and work-related injuries worldwide. They are characterized by localized pain, impaired function, and histopathological changes in tendon structure, which frequently result in chronic disability and lower quality of life [1,2]. The most commonly affected tendons include the rotator cuff, lateral epicondyle, patellar, and Achilles tendons, each associated with considerable healthcare utilization and socioeconomic burden [3].

Exercise-based rehabilitation, particularly eccentric loading programs, is considered the gold standard in the treatment of tendinopathies, with strong evidence supporting its effectiveness in improving pain and function [4,5]. However, a significant minority of patients do not fully recover with exercise alone, prompting physicians to investigate adjuvant therapy targeted at improving treatment outcomes [6].

Dry needling (DN), a minimally invasive method in which small needles are inserted into myofascial trigger points or degenerative tendon tissue, is becoming increasingly popular as a supplemental treatment for tendinopathies [7]. Proposed mechanisms include stimulation of local healing responses, modulation of nociceptive input, and promotion of collagen remodeling, thereby enhancing tendon repair [8]. Several randomized controlled trials (RCTs) have investigated the effectiveness of DN in various tendinopathies, either as a stand-alone treatment or in combination with exercise. While some studies show that DN with exercise improves outcomes more than exercise alone [9,10], others show little or no additional benefit [11,12].

These inconsistent findings highlight the need for a comprehensive synthesis of the available evidence. Therefore, a systematic review and meta-analysis is warranted to evaluate whether combining DN with exercise provides clinically meaningful improvements compared with exercise alone across various tendinopathies. By consolidating existing evidence, this study aims to guide clinicians in adopting evidence-based strategies for tendon rehabilitation and inform future research directions.

## Review

Methodology

This systematic review and meta-analysis was conducted in accordance with the Preferred Reporting Items for Systematic Reviews and Meta-Analyses (PRISMA) guidelines [13]. The study adhered to the principles of the Declaration of Helsinki. As this was a secondary analysis of previously published data, institutional review board approval was not required. The protocol was prospectively registered with the International Prospective Register of Systematic Reviews (PROSPERO; ID: CRD420251130426).

Literature Search

A comprehensive search was performed in PubMed, Cochrane Library, ClinicalTrials.gov, and Google Scholar from database inception to August 13, 2025, with no language restrictions. The search strategy combined Medical Subject Headings (MeSH) and free-text terms related to tendinopathies (e.g., “tendinitis,” “Achilles tendinopathy,” “lateral epicondylitis”), dry needling (e.g., “dry needling,” “intramuscular stimulation”), and exercise therapy (e.g., “eccentric exercise,” “resistance training,” “physiotherapy”). Reference lists of eligible articles and prior reviews were manually screened to identify additional trials.

Eligibility Criteria

Eligible studies were required to (a) be RCTs, (b) include adults (≥18 years) with clinically or imaging-diagnosed tendinopathy, (c) compare DN combined with structured exercise versus exercise therapy alone, and (d) report pain outcomes using a validated scale (Visual Analog Scale (VAS) or Numeric Pain Rating Scale (NPRS)) [14,15], with sufficient data (means and standard deviations) to calculate effect sizes. Reporting of functional outcomes (DASH (Disabilities of the Arm, Shoulder and Hand), PTREE (Patient-Rated Tennis Elbow Evaluation), Tennis Elbow Function Scale) [16-18] was not mandatory for inclusion but was extracted and analyzed where available. Studies were excluded if they were non-randomized, case series, reviews, or conference abstracts without complete data; if they involved pediatric or animal populations; if they lacked comparator groups or statistical details; or if they were duplicate or overlapping publications.

Study Selection and Data Extraction

Titles and abstracts were independently examined by two reviewers, who then evaluated the full text. Disagreements were resolved through consensus or adjudication by a third reviewer. The extracted data comprised study parameters (author, year, country), sample size and demographics, tendinopathy type, intervention specifics (DN procedure, exercise modality), control intervention, follow-up intervals, pain and functional outcomes, and reported adverse events.

Risk of Bias Assessment

The Cochrane Risk of Bias 2 (RoB 2.0) instrument was used to assess methodological quality, which included categories such as randomization, allocation concealment, blinding, outcome data completeness, selective reporting, and other biases [19]. Each study was categorized as "low risk," "some concerns," or "high risk."

Outcomes

The primary outcome of this review was pain reduction, assessed using the VAS [14] or an equivalent validated tool. Pain outcomes were analyzed at four distinct time intervals: immediate (≤1 week), short term (3-4 weeks), mid term (10-12 weeks), and long term (22-24 weeks). Secondary outcomes included improvements in functional status, measured using validated instruments such as the DASH questionnaire, the PTREE, and the Tennis Elbow Function Scale, as well as any reported adverse events related to the interventions [16-18].

Statistical Analysis

Effect sizes were expressed as mean differences (MD) with 95% confidence intervals (CI). A random-effects model (DerSimonian and Laird) was applied to account for heterogeneity. Statistical heterogeneity was assessed using I², Tau², and Chi² statistics, with I² values categorized as low (<25%), moderate (25-50%), considerable (50-75%), and substantial (>75%).

Subgroup analyses were performed according to outcome time frame (immediate, short, mid, long term). Sensitivity analyses were conducted using leave-one-out methods. Funnel plots, Egger’s regression, and Begg and Mazumdar’s tests were used to assess publication bias. Forest plots were generated using RevMan (Review Manager) [20].

Results

Study Selection

**Figure 1:**
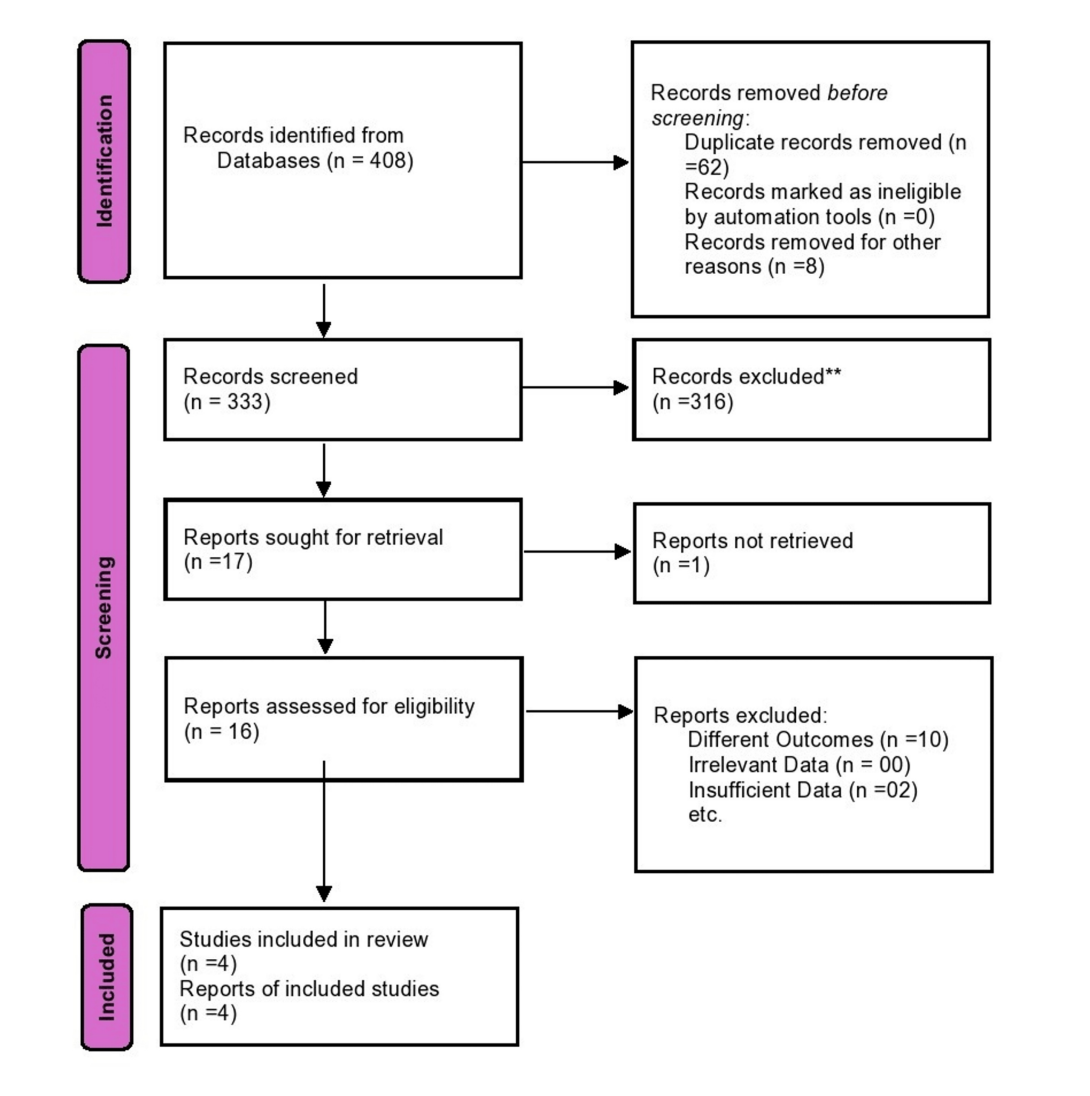
PRISMA Flow Diagram of Study Selection for the Systematic Review and Meta-Analysis PRISMA: Preferred Reporting Items for Systematic Reviews and Meta-Analyses.

The search identified 408 records. After the removal of 62 duplicates and 8 irrelevant records, 333 records were screened. Of these, 316 were excluded at the title/abstract level. Seventeen full-text articles were reviewed, with 13 excluded (10 for unrelated outcomes, 2 for insufficient data, 1 not retrievable). Ultimately, 4 RCTs [21-24] involving 255 patients were included in the meta-analysis, as shown in Figure 1.

*Study Characteristics* Table 1 summarizes study details. Included RCTs evaluated DN plus exercise versus exercise alone in patients with lateral epicondylitis (2 trials), patellar tendinopathy (1 trial), and rotator cuff tendinopathy (1 trial). Sample sizes ranged from 28 to 143 participants. Three studies were single-blind, and one was double-blind. Reported follow-up durations varied from three days to six months.

**Table 1:** Characteristics of Included Studies

Study name	Tendinopathy	Sample size	Study design	Intervention	Control	Reported outcome time
Altas (2022) [22]	Lateral epicondylitis	N_intervention_= 26, N_control_= 26	Single-blind RCT	Dry needling + exercise	Exercise	After 3 weeks and 6 months
Dunning (2024) [21]	Lateral elbow tendinopathy	N_intervention_= 73, N_control_= 70	Single-blind RCT	Dry needling + thrust manipulation + physical therapy	Multimodal physical therapy	1 week, 4 weeks, and 3 months
Royo (2021) [23]	Patellar tendinopathy	N_intervention_= 16, N_control_= 16	Double-blind RCT	Dry needling + exercise	Exercise	After 10 weeks and after 22 weeks
Pourshaffie (2023) [24]	Rotator cuff tendinopathy	_Nintervention_= 14, N_control_= 14	Single-blind RCT	Dry needling + eccentric exercise	Eccentric exercise	3 days

**Figure 2:**
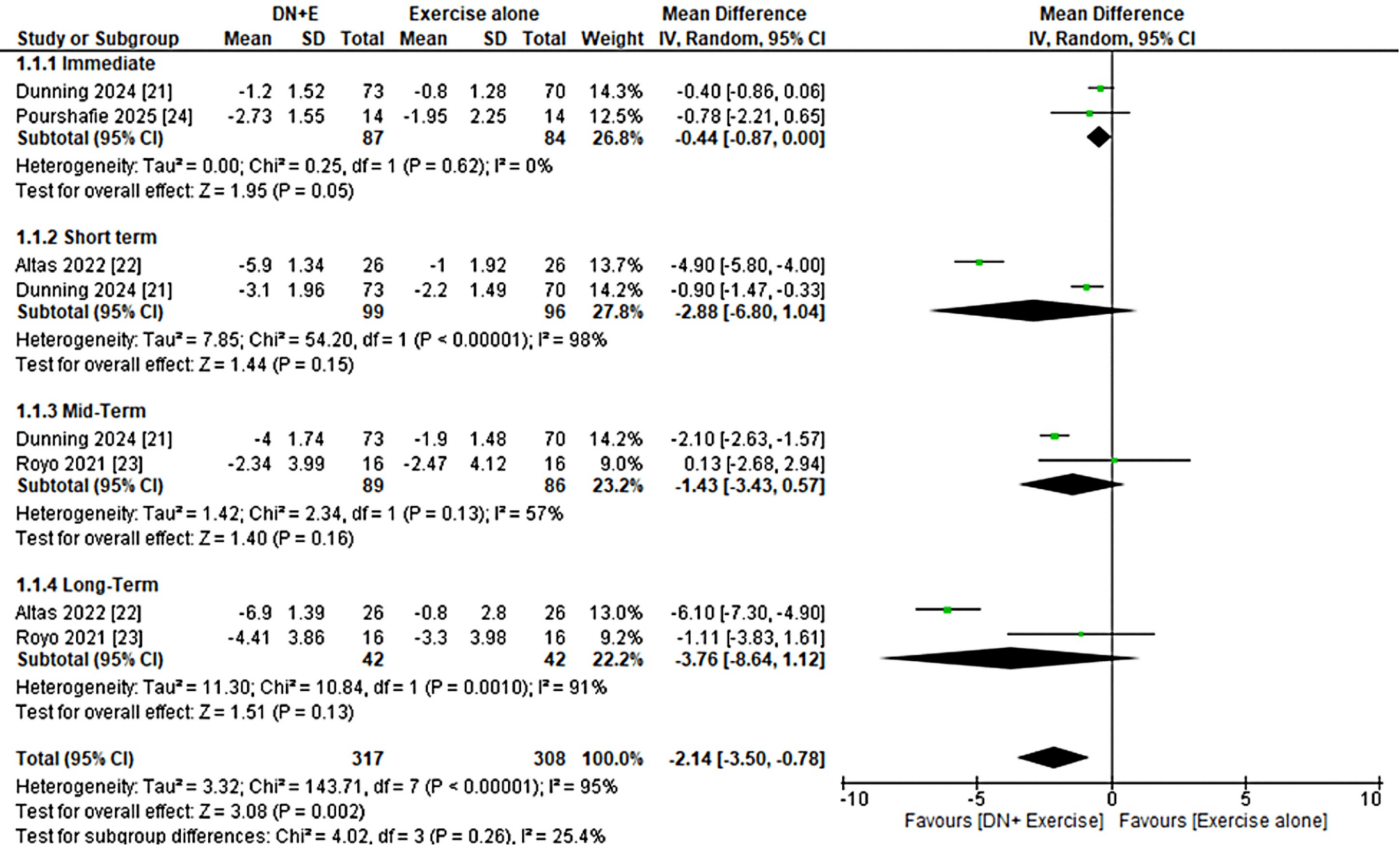
Forest Plot Showing the Effect of Dry Needling Plus Exercise Versus Exercise Alone on Pain Across All Follow-Up Intervals

*Pain Outcomes* Analysis of pain outcomes demonstrated variable effects across different follow-up periods. In the immediate term (≤1 week), DN combined with exercise produced a modest reduction in pain compared with exercise alone (MD = −0.44; 95% CI, −0.87 to 0.00) with no heterogeneity (I² = 0%). At short-term follow-up (3-4 weeks), the pooled effect favored the intervention, though results were not statistically significant, and heterogeneity was substantial (MD = −2.88; 95% CI, −6.80 to 1.04; I² = 98%). Mid-term results (10-12 weeks) also suggested pain reduction (MD = −1.43; 95% CI, −3.43 to 0.57) with considerable heterogeneity (I² = 57%). Long-term outcomes (22-24 weeks) indicated a larger treatment effect (MD = −3.76; 95% CI, −8.64 to 1.12), though heterogeneity remained substantial (I² = 91%). When all studies were pooled, the overall effect favored DN plus exercise (MD = −2.14; 95% CI, −3.50 to −0.78), but substantial heterogeneity was observed (I² = 95%). Subgroup analyses revealed low heterogeneity across time intervals (I² = 25.4%), suggesting that follow-up duration may partly explain differences in treatment effects, as shown in Figure 2.

**Figure 3:**
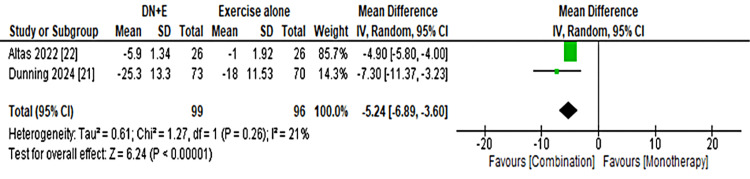
Forest Plot Showing the Effect of Dry Needling Plus Exercise Versus Exercise Alone on PTREE Functional Scores PTREE: Patient-Rated Tennis Elbow Evaluation.

*Functional Outcomes* Functional outcomes were variably reported across the included studies. Two trials assessed the DASH scores [16], but pooling was not possible due to differences in follow-up intervals (3 days vs 3 weeks). Altas et al. observed a greater reduction in disability, favoring the intervention group (−22.7 vs −4.0), whereas Pourshaffie et al. reported comparable improvements between groups. The PTREE [17] demonstrated more consistent findings; pooled analysis at 3-4 weeks showed a significant benefit of DN plus exercise over exercise alone (MD = −5.24; 95% CI, −6.89 to −3.60; I² = 21%), as shown in Figure 3. Dunning et al. further reported sustained improvements in PTREE scores at 1 week, 4 weeks, and 12 weeks. The Tennis Elbow Function Scale [18], also reported by Dunning et al., consistently favored the combined intervention, with greater improvements observed at all measured time points (1 week, 4 weeks, and 3 months).

**Figure 4:**
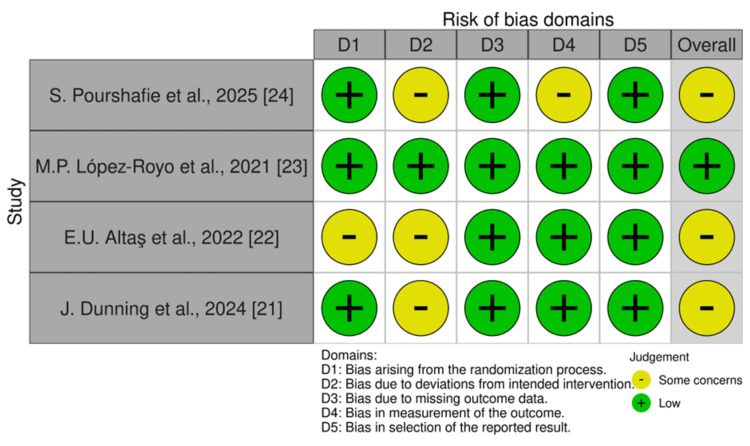
Risk of Bias Assessment of Included Randomized Controlled Trials Using the Cochrane RoB 2 Tool Cochrane RoB 2 Tool.

*Risk of Bias* Risk of bias was evaluated using the Cochrane RoB 2 tool [19]. Among the four included RCTs, one study (López-Royo et al., 2021) [23] was judged to be at low risk of bias across all domains. The remaining three trials (Pourshafie et al., 2025; Altaş et al., 2022; Dunning et al., 2024) [21,22,24] were rated as having “some concerns,” primarily due to issues related to blinding and outcome assessment. Overall, three studies were categorized as having “some concerns,” while one study was considered low risk. No trial was assessed as having a high risk of bias, as shown in Figure 4.

Discussion

This systematic review and meta-analysis pooled data from four RCTs comprising 255 participants to assess the effectiveness of DN combined with exercise compared with exercise alone in the treatment of tendinopathies. The pooled results show that DN with exercise produces moderate but clinically relevant improvements in pain and function, with the highest effects observed at mid- and long-term follow-up. However, substantial heterogeneity across trials highlights the complexity of this intervention and underscores the need for cautious interpretation.

Principal Findings

Our analysis showed that DN combined with exercise reduced pain more than exercise alone, with a pooled MD favoring the intervention (−2.14; 95% CI, −3.50 to −0.78). The effect was mild immediately after treatment but became more noticeable at mid- and long-term follow-ups. Functional outcomes, particularly those assessed using the PTREE [17] and Tennis Elbow Function Scale [18], consistently favored DN plus exercise. These findings support the hypothesis that DN may enhance the benefits of exercise in tendon rehabilitation.

Interpretation in the Context of Included Trials

The included trials evaluated DN across different tendinopathy sites. Altaş et al. [22] found that DN with exercise resulted in superior functional improvements in lateral epicondylitis, whereas Dunning et al. [21] found persistent advantages at 1, 4, and 12 weeks using PTREE and Tennis Elbow Function Scale outcomes. López-Royo et al. [23] found that DN with exercise improved pain and function in patellar tendinopathy at both 10 and 22 weeks, implying long-term benefits. Pourshaffie et al. [24] found equivalent improvements in rotator cuff tendinopathy between groups after only three days, indicating insufficient time for DN-related structural remodeling. These findings suggest that the effectiveness of DN may vary depending on both tendon site and follow-up duration, which is consistent with our subgroup analyses that revealed reduced heterogeneity when stratified by time.

Comparison With Previous Literature

Our findings are comparable with previous systematic reviews, which found that DN can be helpful in lowering musculoskeletal pain; however, most did not focus on tendinopathy [7,25]. Arias-Buría et al. [26] found that DN may relieve short-term pain in musculoskeletal diseases, while Gattie et al. [27] found it useful for chronic pain syndromes. In the context of tendinopathy, Navarro-Santana et al. [28] found DN to be effective for non-traumatic shoulder pain, which is relevant to rotator cuff pathology. However, the evidence using DN as an adjuvant to exercise in tendinopathy has been inadequate. The current study addresses this gap by focusing on RCTs that directly compared DN plus exercise with exercise alone, resulting in therapeutically relevant insights.

Possible Mechanisms of Action

The observed benefits of DN in tendinopathy can be attributed to a combination of neurophysiological and biological mechanisms. At the local tissue level, needle insertion into degenerative tendon tissue is thought to disrupt disorganized collagen fibers and stimulate a localized microtrauma, leading to small hemorrhages and subsequent release of growth factors and cytokines [8,29]. This cascade may upregulate fibroblast activity, promote angiogenesis, and facilitate the synthesis and realignment of collagen fibers, thereby accelerating tendon remodeling and repair [8,29]. These biological effects are particularly relevant in chronic tendinopathy, where degenerative rather than inflammatory changes predominate.

In addition to its local tissue effects, DN exerts important neurophysiological actions. It can modulate nociceptive processing by stimulating A-delta fibers and activating descending inhibitory pain pathways in the central nervous system, thereby reducing peripheral and central sensitization [30]. This mechanism may account for the observed early improvements in pain, even before structural remodeling is fully established.

When combined with exercise, especially eccentric or heavy slow resistance programs, which enhance tendon adaptability, increase tensile strength, and restore load capacity [4,5], DN may act synergistically to optimize both the biological and neurophysiological environment for tendon healing. This dual action likely explains why the greatest improvements in pain and function were observed at mid- and long-term follow-ups, when structural remodeling processes had sufficient time to manifest clinically.

Strengths and Limitations

This systematic review and meta-analysis has several notable strengths. First, we conducted a comprehensive search across numerous databases, including PubMed, Cochrane Library, ClinicalTrials.gov, and Google Scholar, to reduce the risk of publication bias and increase the likelihood of finding all relevant articles. Second, the study adhered to PRISMA principles and used a prospectively documented procedure to ensure methodological transparency and reproducibility. Third, multiple reviewers independently extracted data and assessed risk of bias, with differences resolved by consensus, reducing the possibility of reviewer bias. Importantly, no included trial was classified as having a high risk of bias by the Cochrane RoB 2 tool [19], increasing confidence in the reliability of the available data. Finally, the review examined both pain and functional outcomes across multiple tendinopathy sites, providing a broader clinical perspective than studies restricted to a single tendon disorder.

Despite these strengths, several limitations should be acknowledged. The number of qualifying trials was low (n = 4), and sample sizes within individual studies were limited, reducing the statistical power and precision of pooled findings. The pooled pain outcomes showed significant heterogeneity (I² > 90%), indicating variations in tendinopathy sites (upper vs lower limb), DN methods, exercise regimens, and follow-up duration. Such heterogeneity affects the reliability of pooled results. Functional outcomes were reported inconsistently between trials, and pooling was not possible for measures like DASH [16] due to discrepancies in timing and reporting, making it difficult to draw firm conclusions about functional recovery. Furthermore, the majority of included studies were conducted in single-center settings with very homogeneous demographics, limiting the findings’ applicability to broader clinical practice.

Clinical Implications and Future Directions

The findings indicate that DN may be a beneficial complement to exercise therapy in the treatment of tendinopathies, especially when long-term rehabilitation goals are prioritized. Clinicians should be aware that DN alone is unlikely to provide significant immediate pain relief, although it may improve recovery when combined with regular exercise programs. Given the heterogeneity of current evidence, DN should not replace but rather complement exercise, which remains the cornerstone of tendinopathy management.

Future research should prioritize high-quality multicenter RCTs with standardized DN methods, larger sample sizes, and longer follow-up periods. Trials should stratify outcomes by tendon type to determine whether specific tendons respond better to DN. Furthermore, mechanistic investigations combining imaging and biochemical markers could shed light on the processes through which DN promotes tendon recovery.

## Conclusions

This systematic review and meta-analysis suggest that DN combined with exercise provides modest yet clinically meaningful improvements in pain and function compared with exercise therapy alone in patients with tendinopathies. Although the certainty of evidence is limited by heterogeneity, small sample sizes, and variability in protocols, the findings support the role of DN as a valuable adjunct to exercise-based rehabilitation. Future high-quality multicenter RCTs with standardized protocols and longer follow-up periods are warranted to confirm these results and better inform clinical practice.
